# Poor attention: The wealth and regional gaps in event attention and coverage on Wikipedia

**DOI:** 10.1371/journal.pone.0289325

**Published:** 2023-11-08

**Authors:** Thorsten Ruprechter, Keith Burghardt, Denis Helic

**Affiliations:** 1 Institute of Interactive Systems and Data Science, Graz University of Technology, Graz, Austria; 2 Information Sciences Institute, University of Southern California, Los Angeles, California, United States of America; 3 School of Applied Data Science, MODUL University Vienna, Vienna, Austria; Max Planck Institute for Solid State Research, GERMANY

## Abstract

Wikipedia is an important source of general knowledge covering a wide range of topics. Moreover, for many people around the world, it also serves as an essential news source for major events such as elections or disasters. Although Wikipedia covers many such events, some events are underrepresented and lack attention, despite their newsworthiness predicted from news value theory. In this paper, we analyze 17 490 event articles in four Wikipedia language editions and examine how the economic status and geographic region of the event location affects the attention and coverage it receives. We find that major Wikipedia language editions have a skewed focus, with more attention given to events in the world’s more economically developed countries and less attention to events in less affluent regions. However, other factors, such as the number of deaths in a disaster, are also associated with the attention an event receives. Overall, this work provides a nuanced understanding of attention and coverage on Wikipedia through event articles and adds new empirical analysis to news value theory.

## Introduction

Wikipedia and its many language editions serve as one of the most important crowdsourced platforms of knowledge, with information continuously shared at a global scale [1]. When maintaining these articles, volunteer editors might, however, introduce inaccuracies, hoaxes, or biases [2]. Such flaws distort the represented information and violate Wikipedia’s neutral-point-of-view guideline [3], directly endangering knowledge integrity on Wikipedia [4]. However, flawed content is not the only issue—lack of attention by users and lack of coverage by editors (i.e., knowledge gaps) are equally problematic. These knowledge gaps arise due to innate user preferences to certain topics and popular content. This inequality of coverage and attention harms underrepresented groups [5] and threatens knowledge equity [6], as certain content attracts interested readers who may become editors and in turn contribute new content. Substantial gaps that exclude certain demographics could therefore lead to leaks in the “pipeline of online participation” [7] and produce lasting inequalities in Wikipedia’s information composition that are rooted in the attention particular articles receive.

Unveiling potential inequalities is of high priority to Wikipedia, given the importance of the online encyclopedia as a source of general knowledge around the world. Consequently, past research has investigated certain flaws in Wikipedia’s knowledge and user composition. For example, one of the most prevalent biases on Wikipedia is its gender gap, as both reader and editor demographics [8, 9] as well as article content [10] tend to be biased towards men. Similarly, geographic inequalities can lead to underrepresentation of certain regions [11], possibly due to limited internet coverage and resources for editing. These factors contribute to regional differences in user-generated information that favor some regions over others [12]. Furthermore, the cultural context of a language edition [13, 14] and its editor demographic [15] can also shape what is deemed relevant on Wikipedia [16]. Lastly, given the diversity of Wikipedia’s many language editions, for example in size and popularity [17], rules [18], trends [19], knowledge propagation [20], article quality ratings [21], link structures [22], or topic representation [23, 24] and categorization [25], one should also account for such differences when analyzing inequality on Wikipedia.

In this article, we analyze inequalities across multiple language editions through the perspective of a specific type of Wikipedia content: articles about events taking place all over the world, which can include anything from the death of a queen to strategic victories in Ukraine. Due to the rapid collaboration necessary to update these event articles as news unfold [26], they are subject to contribution patterns that are more dynamic than those for more general, historic content [27]. Additionally, more new editors start editing during these periods [28]. Therefore, rapid contributions to high-attention events could accelerate existing inequalities and biases, which makes it relevant to study why certain event articles attract more attention than others.

**Fig 1:**
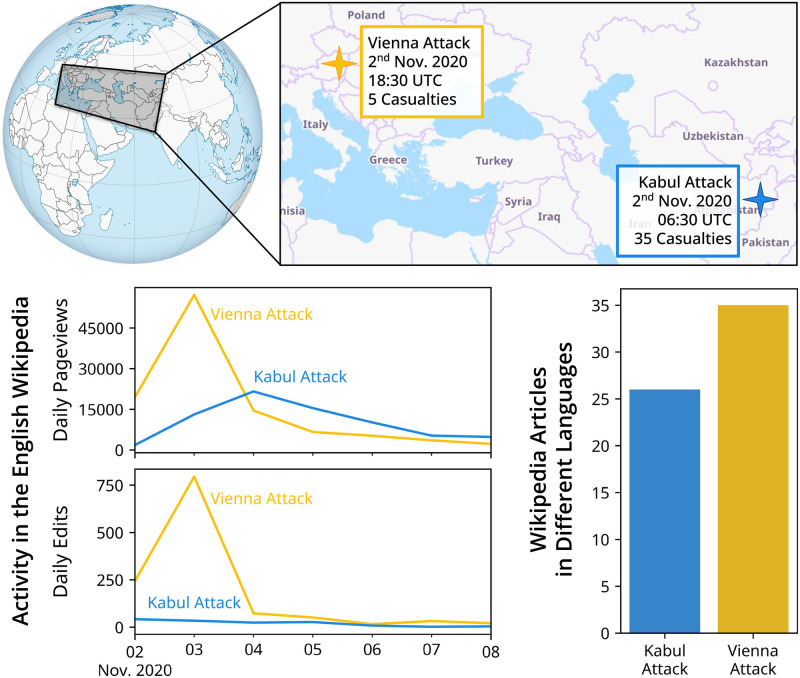
Motivation. On November 2^nd^ 2020, two terrorist attacks occurred: One in Vienna, Austria and one in Kabul, Afghanistan (top). Although both events gained many page views and edits on the English Wikipedia (bottom left), the attack in Austria received much more attention than the one in Afghanistan, including coverage in more Wikipedia language editions (bottom right). Maps retrieved from Wikimedia Commons (top left) and OpenStreetMap (top right).

Fig 1 further motivates our analysis via two articles about events that occurred within twelve hours of each other: The “2020 Kabul university attack” and the “2020 Vienna attack”. Both terror attacks shook the affected communities, with the Kabul attack claiming an even larger amount of fatalities than the Vienna attack. Although both were serious incidents, the English Wikipedia article about the event in Vienna received considerably more attention (page views) and coverage (edits) than the one in Kabul. Moreover, by 2022, fewer Wikipedia language editions have a dedicated article on the Kabul attack than on the Vienna attack (26 and 35, respectively). Besides the seriousness of the two events, additional factors may have contributed to their respective newsworthiness, leading to the apparent skew in attention and coverage.

What makes an event newsworthy is an important question in communication and journalism studies [29]. Early literature proposed taxonomies of “news values” that serve as indicators of newsworthiness [30], which have been revised repeatedly to match the changing patterns of news over time [31]. In regard to online communities and social media, Lin et al. [32] investigate how media attention affects shared attention on Twitter, while West et al. [33] measure the popularity public figures receive after their death. Furthermore, Robertson et al. [34] find that negative sentiment in online news articles leads to higher reader engagement. More relevantly, news value theory has also been applied to Wikipedia [35], as its numerous language editions cover important current and past events [26, 36]. This includes various events of public interest, for example the Zika epidemic [37] or COVID-19 pandemic [38]. Events with negative connotations generally draw a lot of attention to Wikipedia, as researchers have used the platform to study the public’s response to natural disasters [39], terrorist attacks [40], regional conflicts [41], or plane crashes [42]. Additionally, previous research highlighted that media coverage [43], geographical proximity and socio-economic factors [40], as well as article category [44] could influence the attention articles receive and that news value theory could provide additional insight into these relationships [35]. Nonetheless, why certain Wikipedia event articles receive high or low attention is still not totally clear. Analyzing these correlations between specific factors and the attention Wikipedia viewers or editors pay to particular events is essential to better understand potential persistent inequalities as well as skewed attention or coverage on Wikipedia [5].

In this work, we focus on two possible factors associated with heterogeneous attention and coverage in event articles: economic power and geographic region of the event’s location. Accordingly, we aim to answer the following research questions:

- **RQ1:** How does the skew in attention or coverage relate to the economic power or geographic region of the country an event occurs in?
- **RQ2:** How are these associations mediated by article language?

To this end, we investigate 17490 Wikipedia articles that cover events between 2016 and 2020 across four large language editions: English and Spanish, which are more globally spoken, as well as the more localized German and Italian Wikipedia editions. We first leverage Wikidata and Wikipedia APIs as well as external data sources to build our list of event articles and collect event metadata. Next, we examine descriptive statistics about the collected event articles and find that a considerable number of articles does not receive any attention or coverage in the first week after they happen and that of those that do, many occur in higher income countries. Afterwards, we employ features grounded in news value theory, the theory used to understand what news receive attention, to train state-of-the-art machine learning models from which we compute the impact of economic power and geographic region on event attention. We find that the existence and popularity of articles can be highly skewed towards richer countries across Wikipedia language editions. That said, these effects are mediated by the type of event, whether sports, disaster, political, or cultural events, where attention to disaster events is more strongly positively correlated with the wealth of the country in which an event occurs. Altogether, this work (i) sheds light on potential biases in Wikipedia, and (ii) provides empirical evidence of news value theory within a popular multi-lingual platform. Lastly, we provide our dataset, models, and experimental code on GitHub (https://github.com/ruptho/wiki-event-bias).

## Materials and methods

### Mining events and their features

We leverage Wikidata to collect our candidate list of events [45]. We choose Wikidata as it is a general knowledge base that does not focus on a specific event type—as other databases do with military or political events [46]—and can be readily analyzed. Moreover, as the Wikimedia Foundation manages both Wikidata and Wikipedia, Wikidata acts as a central storage for Wikipedia metadata, directly providing links from Wikidata entities to Wikipedia articles.

To answer our research questions, we enrich event information from Wikidata with external metadata, such as economic or geographic information provided by The World Bank [47] to build features that lend from news value theory and research on Wikipedia news event articles.

#### Collecting event articles

We utilize Wikidata [45] to collect events that occurred between 2016 and 2020. Wikidata collects information about entities (e.g., people or events) in the form of “items” which contain “statements”. In the case of events, such statements describe, among others, time and country the event occurred in. As an example, for the “2020 Kabul University attack”, these values are, respectively, “Afghanistan” and “2 November 2020” (see https://w.wiki/6RLk).

**Retrieving events from Wikidata.** To build our list of events, we use the Wikidata API [48] to retrieve all items that have a “point in time” and “country” statement, making these the prime indicators for an item being an event (i.e., something important that happened at a specific time). We utilize SPARQL queries to retrieve 56877 event items from Wikidata including their Wikidata ID, name, country, and relevant dates (“point in time” as well as “start date” and “end date”, if existent). We set the actual event date to the value of the “start date” statement if that statement is available and use “point in time” otherwise. We ignore events that have multiple “country” or “point in time” statements on Wikidata. After initial investigation of our data, we remove events with January 1^st^ as the set event date, as often times these entries are either faulty or include year-long events as well as summaries of seasons by sports teams and leagues (e.g., “2016 Kyrgyzstan League”).

**Measuring attention to event articles.** For each Wikidata event item, we retrieve the corresponding URL to the Wikipedia article in English, Spanish, German, and Italian. We choose this list of Wikipedia editions as they contain two languages spoken widely across the globe (English and Spanish) and two languages that are more localized to their country of origin (German and Italian). This allows for analysis of how both “global” (spread out across multiple continents) and “local” (focused on a single region or country) communities respond to external events. Additionally, the authors are proficient across these four languages, which facilitates our understanding of the article contents for this work. After retrieving the URL for all Wikipedia articles and dropping those for which no article could be found, we utilize the Wikimedia API [49] to collect more metadata about events and their corresponding articles, for example article title, the Wikipedia categories it belongs to, and the first paragraph of the article. Futhermore, we define the attention an event article receives in a given Wikipedia language edition with the overall pageviews (or edits) the event article and its redirect pages [50] received within the first seven days after the event date, and collect both pageviews and edits using the Wikimedia API. As robustness checks, we also compare our retrieved amount of views and edits within seven days of an event with those within thirty days and find that they are highly correlated (Spearman’s *ρ* = 0.96 for views and *ρ* = 0.94 for edits). Additionally, we further compute the maximum daily page views (edits) in the first seven days after an event, which again are strongly correlated to the seven-day views (Spearman’s *ρ* = 0.98) and edits (Spearman’s *ρ* = 0.95). Therefore, we consider the views and edits an event article received within the first week after it occurred as our main measures of attention and coverage.

#### News features of Wikipedia events

To investigate event article attention and coverage, we lend from existing news value taxonomies that have included factors such as whether an event brings good or bad news, contains references to powerful individuals or organizations, affects similar demographic groups as the target audience, or impacts many people [31]. We also consider findings from research about news in the English Wikipedia [35, 36] to conceive features relevant to our research questions. Overall, we devise nine event article features:

**Event category.** The category of the event article, which is either sports, politics (e.g., elections, peaceful protests, speeches), disasters (e.g., natural disasters, plane crashes, shootings), or culture (e.g., award shows).

**Geography.** The geographic region the event happened in (East Asia & Pacific, Europe & Central Asia, Latin America & Caribbean, Middle East & North Africa, North America, South Asia, or Sub-Saharan Africa), c.f. The World Bank [47] for mapping between country and geographic region. While this categorization does not differ much from the breakdown of countries by continent, it does provide a more accurate split that better captures regional affiliation. This models “Proximity” as it occurs in news value taxonomies.

**Economy.** This feature set models the concept of “Power Elite” as described in news value theory in terms of economic power of an event country.

- *Gross Domestic Product per Capita (GDP_pc_).* The event country’s *GDP*_*pc*_ in dollar.
- *Income Class.* The event country’s income categorized via the gross national income per capita (low: < $1 046, lower mid: $1 046—$4 095, upper mid: $4 096—$12 695, high: > $12 695).

**Magnitude.** The number of people possibly affected by this event, approximated by the event country’s population.

**Uniqueness.** This feature set combines the concept of “Follow-Up” and “Uniqueness” of news value theory.

- *Country Uniqueness.* Event articles in the same country and Wikipedia in the last 30 days (log scale).
- *Category Uniqueness.* Event articles in the same event category and Wikipedia in the last 30 days (log scale).

**Relevance.** The relevance of the language edition in the event country as the median monthly views from the country to the language edition in the five months prior to the event. Due to Wikipedia’s global popularity [1], we employ this metric as a proxy for measuring digital literacy (and thus internet penetration) of a country. For example, we find that the number of internet users per country [51] strongly correlates with *Relevance* for all event article countries recorded in the English Wikipedia (Spearman’s *ρ* = 0.71). Additionally, internet penetration per capita is highly correlated with socio-economic indicators such as *GDP*_*pc*_ (Spearman’s *ρ* = 0.82). For *Relevance* as well as *Prominence* (below), we also compute metrics for three and seven months and find high correlations between using three, five, and seven months (Spearman’s *ρ* > 0.98 for all comparisons), so we stick with the five-month timespan for both metrics.

**Prominence.** The prominence of the event country in the language edition as the median daily views of the country’s main article in the five months prior to the event. This metric reflects the approximate importance of the event country to the general visitor population of a particular language edition, such as the general presence of the country in media consumed by the target audience.

To retrieve features derived from Wikipedia metrics (e.g., *Relevance* and *Prominence*), we utilize the Wikimedia REST API [49]. Additionally, we collect economic and geographic data from The World Bank [47]. Finally, for classification of events into event categories we first consider the articles’ categories in the corresponding Wikipedia editions and then match the articles’ Wikipedia categories as well as the articles’ titles to language-specific keyword lists (see data on GitHub). For articles containing multiple keywords, we select our event category with the most matches. In case no keyword matches were found (361 events), two of the authors manually labeled these remaining events and agreed on event categories for all but 51 events (e.g., Lunar or Solar eclipses), which were consequently removed from analysis. We further apply an unsupervised approach to detect possible topical article categories using word embeddings and K-means clustering. To this end, we use the Wikimedia API to retrieve articles’ first paragraphs and extract multi-lingual SentenceTransformers embeddings [52] for the first 50 words of each article. We then perform K-means clustering on these embeddings for 2 to 100 clusters and find the best-fitting cluster size with the highest silhouette score at 9. After extracting words with the highest TF-IDF scores for each cluster, we observe that clusters largely adhere to our initial labeling, as we find clusters congruent to our labels: (i) cultural events, festivals, and award shows (*culture*) (ii) natural disasters, plane crashes, terrorist attacks, and murders (*disasters*) (iii) elections in the English Wikipedia (*politics*) (iv) elections in other language editions (*politics*) (v) tournaments, championships, and Olympics in the English Wikipedia (*sports*) (vi) tournaments, championships, and Olympics in other language editions (*sports*) (vii) cycling and other non-motor races (*sports*) (viii) motor sports such as Formula 1 (*sports*), and (ix) tennis or other similar tournament sports (*sports*). Therefore, we stick to our initial categorization of articles into sports, politics, disasters, or culture for the event category feature.

After all processing steps, we match the initial list of 56877 Wikidata items to 17490 Wikipedia articles (7921 English, 3278 German, 2737 Italian, 3554 Spanish).

### Measuring effects on attention

To measure how economic and geographic factors influence attention to event articles, we train XGBoost models (eXtreme Gradient Boosting) [53] and estimate the effects via local explanations of attention using SHAP values [54].

#### Model setup

We train the XGBoost models for two machine learning tasks predicting (i) attention and (ii) coverage. Both tasks consist of two subtasks: A classification subtask to predict whether articles receive any attention (> 10 views) or coverage (> 0 edits) and a regression subtask to predict the level of either attention (views) or coverage (edits). For the regression, we use the logarithm of views and edits as the target variable to better depict the scale of attention, as, for example, the difference between a hundred or a thousand views is more meaningful than between ten or eleven thousand.

For both subtasks, we utilize the features defined in Section “News Value Features”, but only use *GDP*_*pc*_ instead of income class as the feature bearing economic information due to high correlation across all countries (Spearman’s *ρ* = 0.95). We also exclude geographic region because of its relation to *GDP*_*pc*_ (Kruskal-Wallis *p* < 0.01) and instead use region to group resulting SHAP values for visualization. Furthermore, we add a categorical variable to distinguish between separate language editions and perform one-hot encoding for all categorical features. Finally, we fit XGBoost models for both classification and regression tasks.

Regarding data, the classifiers use all 17490 event articles (7921 English, 3278 German, 2737 Italian, 3554 Spanish). The view regressors are trained on the 12712 articles (6670 English, 1664 German, 1573 Italian, 2805 Spanish) that received more than 10 views, while the edit regressors are trained on 10495 articles (5725 English, 1543 German, 1292 Italian, 1935 Spanish) that were edited at least once.

For all our experiments, we split the corresponding five-year dataset (2015–2020, 60 months) into three temporally sequential sets. We use training (2016–2018, 36 months) and validation sets (2019, 12 months) for fine-tuning and hyperparameter search (see GitHub) and evaluate the models using F1-score (classification) and mean squared error (regression). We select the best-performing model regarding the train-validation split and use a hold-out test set (2020, 12 months) to assess model performance. Finally, we retrain the best-performing model using the full corresponding dataset and compute SHAP values for all features.

#### Model performance

The XGBoost classifier predicting whether an article receives >10 views (>0 edits) reaches an F1-score of 0.867 (0.726) on the test set. As robustness experiments, we fit logistic regressions, support vector classifiers, and Random Forest classifiers. We find that according to the performance on the test set XGBoost performs better or is comparable to the alternative models (95% bootstrapped confidence intervals).

Furthermore, the XGBoost regression models attain a mean squared error on the test set of 3.95 for views and 1.56 for edits. We also perform robustness checks for this task using negative binomial regression, support vector regression, and Random Forest regression. Even though negative binomial regression—as a generalization of linear regression to cardinal numbers—typically has an advantage over other methods because of its explanatory power, we find that XGBoost significantly outperforms the negative binomial (95% bootstrapped CI). We observe no difference in the test error between XGBoost and the other non-linear approaches.

#### Estimating effects on attention via SHAP values

SHAP values are a model-agnostic measure of feature importance based on the game-theoretic principle of Shapley values [54]. In general, SHAP values measure the contribution of features to the individual predictions of a model computed through feature permutation. They enable interpretation of local explanations and marginal contribution of features, which distinguishes them from classical measures of effect size such as negative binomial or linear regression coefficients, which interpret only global effects. However, SHAP values can also combine local explanations to better understand global model structure. In our case, we are interested in specific effects of discrete data points or groups (i.e., events or event types). Therefore, the ability to combine local and global explanations as offered by SHAP values is a more principled approach to estimating effects than alternative effect size estimators, such as regression coefficients. Moreover, our robustness analysis showed that non-linear models outperform linear regression models, so we can not make conclusive statements based on linear models. Finally, SHAP values make no assumption about feature independence—another limitation of classic dependency metrics—and can estimate feature interaction effects.

In practice, SHAP values measure how feature values affect the model’s prediction, relative to a baseline. For binary classification, the baseline is the log odds of positive (true) samples and for regression, it is the average of the target variable. While we interpret values for continuous features as provided by SHAP, for each categorical feature we sum SHAP values of the binary features generated by one-hot encoding and obtain a single value to understand the total contribution of the original categorical feature. We also compute ACV Shapley values [55], which do not change our overall findings.

Although SHAP values do not assert causality, they help interpret black-box models and provide insights into the importance of many potential mediating features. While we can not control for all mediators and confounders, by carefully selecting features in light of news value literature, we test many mediators proposed by theory. In other words, by including socio-demographic (e.g., relevance and prominence) and event-based (e.g., uniqueness) mediators as a baseline [56], we estimate the excess attention and coverage associated with *GDP*_*pc*_ and geographic region.

## Results and discussion

### Descriptive analysis of event articles

#### Attention by category and income

We characterize our 17490 event articles by language edition, category, income class, and whether they receive attention in the first week of the events’ occurrence. To reduce noise, we only consider event articles with more than ten views (a proxy of attention) or at least one edit (a proxy of coverage). We summarize the data statistics in Table 1, and provide p-values for statistical tests throughout the text. Please note that, in all section that follow, we will consistently report Bonferroni-corrected p-values based on a significance threshold *α*/*n*, where *α* = 0.05 and *n* represents the number of tests conducted.

**Table 1:** Dataset statistics. We show our dataset by Wikipedia language edition, category of event articles, and income class of the country the events happened in. H = High, UM = Upper mid, LM = Lower mid, L = Low.

	Article Category					Income Class				
	Name	Overall		>10 Views	>0 Edits	Name	Overall		>10 Views	>0 Edits
		#	%				#	%		
English	Sports	4194	52.9%	86.3%	73.0%	H	5378	67.9%	84.5%	71.3%
	Politics	2017	25.5%	87.2%	75.5%	UM	1611	20.3%	86.2%	77.7%
	Disaster	984	12.4%	74.2%	70.1%	LM	765	9.7%	79.2%	67.2%
	Culture	726	9.2%	77.5%	61.8%	L	167	2.1%	80.2%	76.0%
	**Sum**	**7921**	**100%**	**84.2%**	**72.3%**	**Sum**	**7921**	**100%**	**84.2%**	**72.3%**
German	Sports	2499	76.2%	42.8%	39.1%	H	2044	62.4%	57.5%	52.9%
	Politics	399	12.2%	76.4%	70.9%	UM	897	27.4%	37.2%	34.8%
	Disaster	264	8.1%	75.8%	76.1%	LM	277	8.5%	42.2%	40.1%
	Culture	116	3.5%	76.7%	69.8%	L	60	1.8%	61.7%	63.3%
	**Sum**	**3278**	**100%**	**50.8%**	**47.1%**	**Sum**	**3278**	**100%**	**50.8%**	**47.1%**
Spanish	Sports	2055	57.8%	86.6%	58.8%	H	2238	63.0%	79.7%	52.4%
	Politics	777	21.9%	69.1%	47.6%	UM	1007	28.3%	81.7%	61.5%
	Disaster	466	13.1%	62.9%	50.4%	LM	216	6.1%	66.2%	46.3%
	Culture	256	7.2%	76.2%	47.7%	L	93	2.6%	60.2%	46.2%
	**Sum**	**3554**	**100%**	**78.9%**	**54.4%**	**Sum**	**3554**	**100%**	**78.9%**	**54.4%**
Italian	Sports	2053	75.0%	60.1%	48.5%	H	1803	65.9%	58.7%	48.2%
	Politics	363	13.3%	42.7%	38.3%	UM	744	27.2%	60.5%	50.1%
	Disaster	163	6.0%	49.7%	46.6%	LM	143	5.2%	32.2%	26.6%
	Culture	158	5.8%	65.2%	51.9%	L	47	1.7%	38.3%	25.5%
	**Sum**	**2737**	**100%**	**57.5%**	**47.2%**	**Sum**	**2737**	**100%**	**57.5%**	**47.2%**
**All**		**17490**	**100%**	**72.7%**	**60.0%**	**All**	**17490**	**100%**	**72.7%**	**60.0%**

In our four language editions, we find that most articles are viewed within the first week of the event, while the number of edited articles is slightly smaller (cf. Table 1, columns “>10 Views” and “>0 Edits”). Specifically, event articles in the global English and Spanish Wikipedia editions are considerably more likely to be viewed or edited than the German or Italian editions (chi-square tests, *p* < 0.025). There are also differences in attention and coverage between language editions when partitioned into the four article categories: sports, politics, disasters, or culture (chi-square tests, *p* < 0.0125). For example, German has a relatively large number of sports articles, but less than half of them are viewed or edited as opposed to, e.g., English where more than 73% of sports articles are viewed or edited. Additionally, politics and disasters are less likely to be viewed or edited in Italian than German, Spanish, or English (chi-square tests, *p* < 0.0083). Furthermore, in all languages users are more likely to view or edit articles from countries in the upper-middle or high-income classes within the first week of an event’s occurrence, and fewer from low-income countries (cf. Table 1, columns for income class), although there is no significant difference for German (chi-square tests, *p* > 0.0125). In Spanish, although more articles about events in high-income countries exist, events in upper-middle income countries are more likely to be viewed or edited (chi-square tests, *p* < 0.025), as many Latin American countries are in this income class.

When focusing on disasters, much like the Vienna and Kabul attacks seen in Fig 1, lower and lower-middle income countries are more likely to have articles (chi-square tests, *p* < 0.0125) and these articles are often viewed or edited within one week. This may be due to heightened news coverage or the relevance of the event to our Wikipedias’ demographics. In contrast, the investigated Wikipedia editions rarely cover political or cultural events in lower or lower-middle income countries.

Altogether, we show that Wikipedia editions differ in the numbers of articles per category. The differences we find are less pronounced between global (English and Spanish) and local (German and Italian) language editions. Our event data indicates that the number of event articles is skewed towards countries of higher economic status.

#### Geography and attention to events

**Fig 2:**
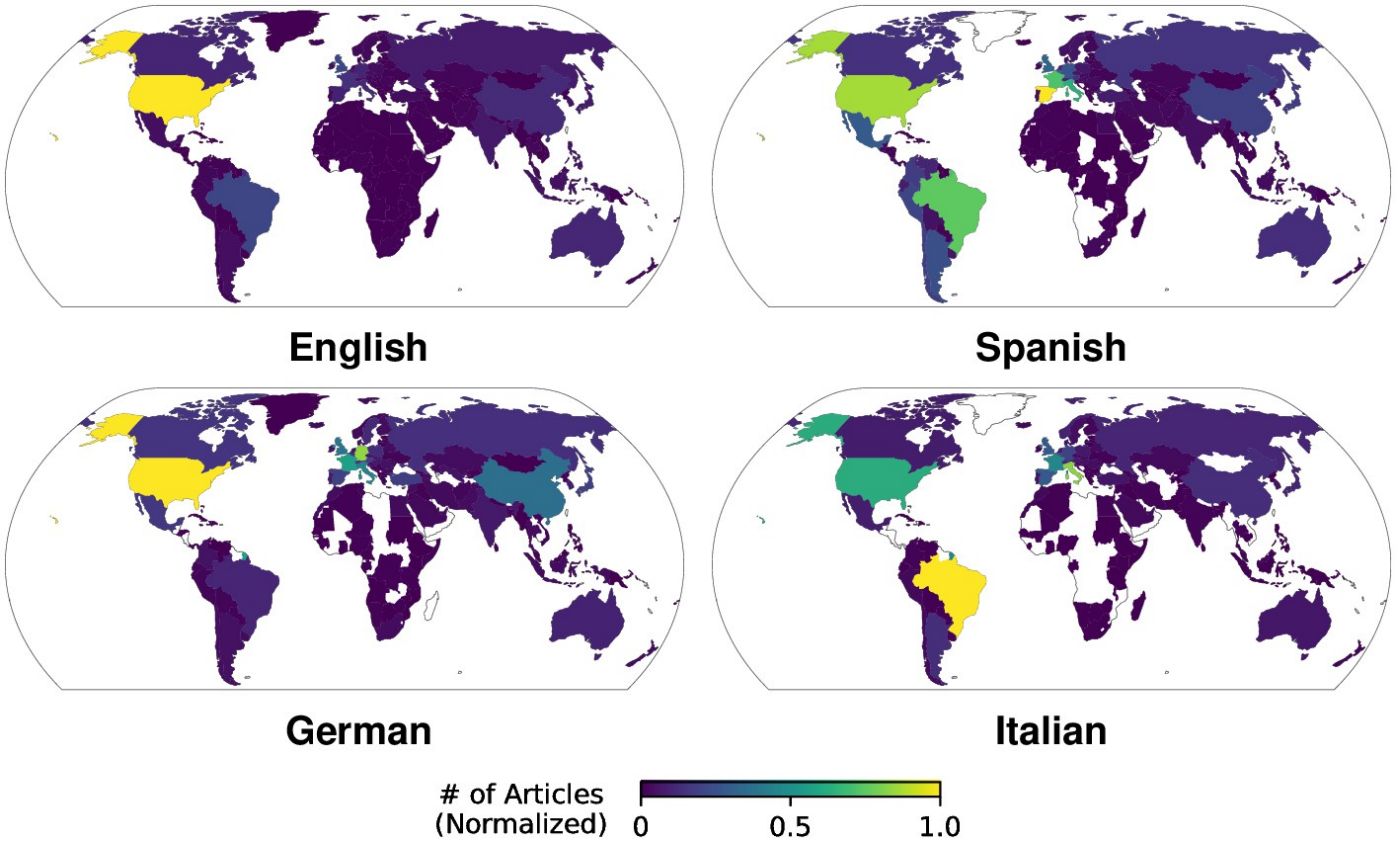
Number of event articles per country. We visualize the number of articles about events in a country normalized by the highest country value for each language edition. The number of events is strongly skewed towards regions which contain the language edition’s native speakers and the USA in general. White areas span countries with no event articles.

In Fig 2 we show the number of articles, which demonstrates that the global English Wikipedia strongly focuses on Anglophone countries, as 30.3% of event articles relate to events in the USA or United Kingdom. In the Italian and German Wikipedia we observe increased numbers for events in Europe and the USA in the Italian and German Wikipedias (54.4% and 8.3% as well as 46.1% and 9.4% of event articles, resp.). Additionally, Italian exhibits significantly higher number of articles related to Brazil than other language editions (11.2% of event articles; Z-tests, *p* < 0.0167) mainly driven by the 2016 Summer Olympics in Rio de Janeiro. The Spanish edition, meanwhile, has many events from the US (14.3% of articles) and significantly more articles from Hispanophone countries, such as Latin America and the Caribbean or Spain (23.1% and 13.9% of all articles), than all other languages (Z-tests, *p* < 0.0083).

**Fig 3:**
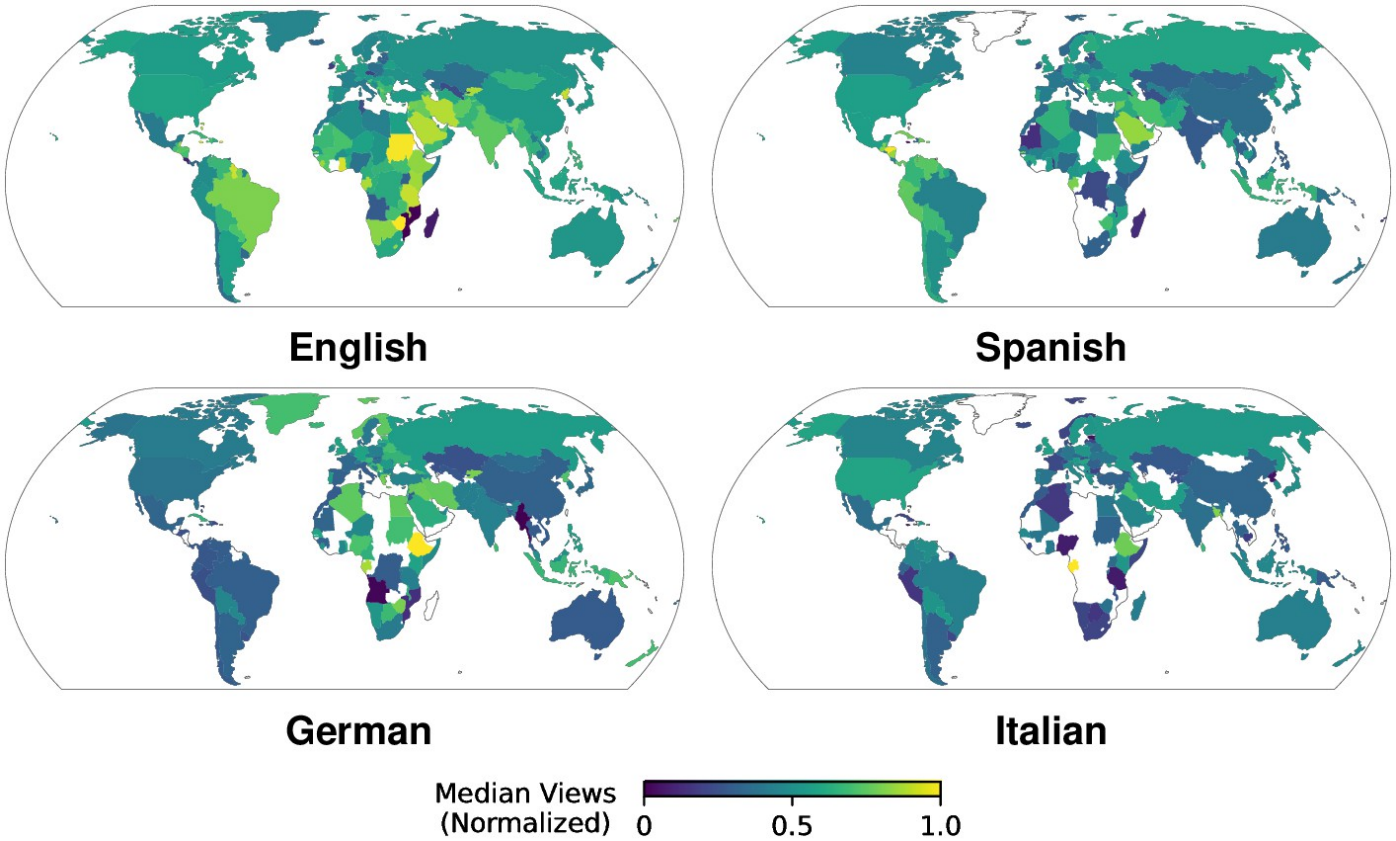
Median views to event articles per country. We show median views to articles about events in a country normalized by the highest country value for each language edition. Median views are more equally distributed and sometimes even high in countries with non-native speakers. White areas cover countries without event articles.

In contrast to the number of articles, we observe no clear regional focuses for the median article views by country (Fig 3). Most notably, for English, German, and Italian the countries with the highest relative median views are those with a smaller number of articles and are all located in Sub-Saharan Africa (Sudan, Ethiopia, and Gabon, respectively). However, in general, we find higher values for views to events in countries where the language of the Wikipedia edition is an official language compared to those where it is not (Mann-Whitney U tests, *p* < 0.0125). For example, in the Spanish Wikipedia we find the highest median views for Honduras.

We conclude that there is a clear surplus in the number of articles in countries relevant to the user demographics of the Wikipedia editions (either in proximity, cultural context, or language), and that we observe an increased level of attention to event articles in countries relevant to the Wikipedia language, although highest median attention is registered for countries relatively foreign to the language edition.

### Skew in event article attention

We now investigate SHAP values for our classification and prediction tasks to measure the effect of certain factors, such as *GDP*_*pc*_, on the prediction outcome with respect to the geographic location of events. In this work, we consider other news value features besides *GDP*_*pc*_ as control and, besides article category, exclude visualization of SHAP values for these features. We hereafter only elaborate on the results for predicting attention and exclude those for coverage, as findings for attention and coverage are qualitatively similar in both the classification and regression subtasks. All results not included in this paper are available on GitHub.

#### Effect of *GDP*_*pc*_ on whether articles receive any attention

**Fig 4:**
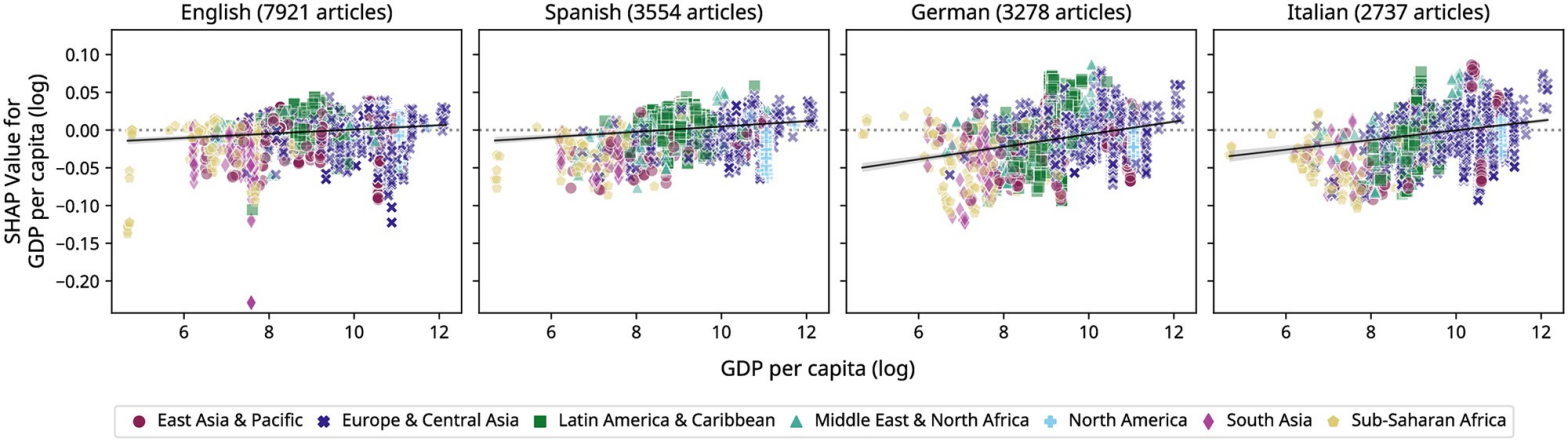
SHAP values for *GDP*_*pc*_ for predicting whether events receive attention. We show SHAP Values for *GDP*_*pc*_ for all articles by language and geographical region for our XGBoost classifier predicting whether articles receive more than ten views. For this classifier, SHAP values for *GDP*_*pc*_ (y-axis) measure by how much corresponding values for *GDP*_*pc*_ (x-axis) change the base prediction, i.e., the average log-odds of 0.36. We find a positive upward trend for SHAP values illustrated by linear regression lines, indicating that higher *GDP*_*pc*_ correlates with higher likelihood of events receiving any attention.

We first investigate how wealth might affect whether an event article receives any attention at all via our trained classification model. Fig 4 visualizes SHAP values for *GDP*_*pc*_, demonstrating the impact of *GDP*_*pc*_ on the XGBoost classifier’s prediction for all events across separate geographic regions. We observe an upward linear trend for *GDP*_*pc*_ and its SHAP values, which indicates that events in richer countries are more likely to be viewed within one week of the event occurring. Notably, this positive correlation is stronger for the local German and Italian editions (Pearson’s *r*, *r* = 0.30 and *r* = 0.20, resp., *p* < 0.0125) and slightly lower for the global English and Spanish editions (Pearson’s *r*, *r* = 0.14 and *r* = 0.17, resp., *p* < 0.0125). According to our XGBoost classifier, the probability of events receiving at least some attention steadily increases with rising *GDP*_*pc*_.

Moreover, we can identify and characterize outliers in our prediction task through the local explanations provided by SHAP values. For example, in English we observe that a number of events in South Asia, which by closer investigation are Badminton tournaments in India, have the lowest SHAP values for *GDP*_*pc*_ out of all observed events (−0.23). Furthermore, the effect of *GDP*_*pc*_ on prediction outcome differs across events in Sub-Saharan Africa, as for example in German values for events in some lower-income countries in Africa (e.g., Somalia) are higher than for other richer countries in the region (e.g., South Africa). Additionally, lower values for events in certain high-income countries in Europe and Central Asia, observable for example in English or Italian, can be explained by these articles containing recaps of non-prominent sports events (e.g., bicycle races) that were created well after the event took place.

#### Effect of *GDP*_*pc*_ on the level of attention

**Fig 5:**
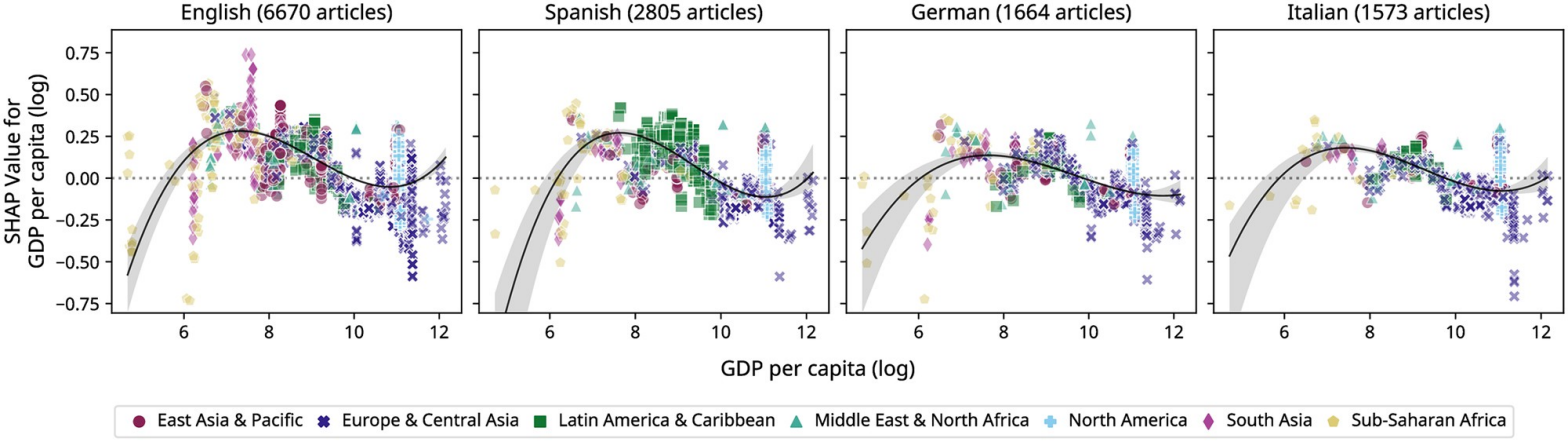
SHAP values for *GDP*_*pc*_ for predicting the level of attention. We show SHAP Values for *GDP*_*pc*_ for all articles by language and geographical region for the XGBoost regressor predicting the number of views (logarithmic scale). For this regressor, SHAP values for *GDP*_*pc*_ (y-axis) measure by how much corresponding values for *GDP*_*pc*_ (x-axis) change the average prediction (i.e., average views in logarithmic scale of 7.721 for this regression task). We observe non-linearity in the relationship between SHAP values and *GDP*_*pc*_, as illustrated by polynomial regression lines of third degree, suggesting that other factors besides *GDP*_*pc*_ influence prediction results.

To analyze the potential influence of *GDP*_*pc*_ on the level of attention in views for the articles that received at least some attention (>10 views), we now consider the trained regression model. For this XGBoost regressor, we observe no clear visual pattern for SHAP values for *GDP*_*pc*_ across all language editions (Fig 5; see S1 Table for pairwise significance tests). Rather, we find an overall negative correlation between *GDP*_*pc*_ and its SHAP values for all four language editions (Pearson’s *r*: −0.55 for English, −0.60 for Spanish, −0.50 for German, −0.51 for Italian; *p* < 0.0125). Even though these findings suggest higher attention for lower-income regions, they may be counterintuitive in that only the most prolific events even surpass the threshold to receive attention or be covered on Wikipedia, while less consequential events are left out completely. While non-native editors might simply not show interest or be aware of such events, local editors may not have the time and resources to edit their native language Wikipedia [11], let alone work on larger language editions (e.g., English) to increase their representation in these vast knowledge bases. Consequently, certain regions in the Global South receive less attention from both non-native and native Wikipedia visitors in the corresponding language editions. This is one reason Wikipedia suffers from regional differences in user-generated information that favor regions in the Global North over regions with less economic power in the Global South [12]. These patterns are consistent with the findings previously discussed in Fig 2, which suggested that higher median attention to articles about events in a particular country might be inversely related to the amount of articles about events in that country.

On a separate note, events in certain countries receive more or less attention than would be expected from their *GDP*_*pc*_ or region alone. For example, while we observe lower SHAP values for events in countries with lower *GDP*_*pc*_ (i.e., in Sub-Saharan Africa), especially for the English Wikipedia a considerate amount of events occurring in certain lower income countries, such as South Africa or Nigeria, exhibit larger positive SHAP values. Next, events in South Asia (e.g., India or Pakistan) receive more attention than most other events in all Wikipedia editions, a pattern that is again more profound in English and underscores this language edition’s global presence.*GDP*_*pc*_ SHAP values of events in Latin America and the Caribbean are relatively high in the Spanish Wikipedia, especially when compared to German and Italian. This is even more pronounced for the XGBoost model predicting coverage (see GitHub), suggesting that the Spanish Wikipedia focuses more on this region. Furthermore, events in richer countries in Europe and Central Asia generally do not have positive values associated with them, indicating that *GDP*_*pc*_ alone does not determine much about which events receive more attention, at least for this region. For example, local elections or sports events in Switzerland, such as competitions in the 2020 Winter Youth Olympics, generally receive very low attention, even though Switzerland’s *GDP*_*pc*_ is very high. In the case of North America, analyzing the effect of *GDP*_*pc*_ on predictions produced by our models indicates a split picture: On the one hand, certain events, such as motor races or mayoral elections receive less attention, even when located in high-income countries such as the USA. On the other hand, presidential elections and popular award shows such as the Oscars increase attention in all language editions. Multiple of these examples suggest that other factors, such as geographic proximity to a language edition’s main user demographic or article category have a stronger influence on the attention events receive. These findings are akin to social impact theory [57], in which social, cultural, or spatial proximity between the source and targets is one of the defining factors for the relevance of an event to an audience. Overall, while large sporting events or reoccurring local elections generate articles that receive at least some attention or coverage, they do however not receive as many views or edits as rarer high-attention events such as award shows or articles in regions where an edition’s language is widely prevalent.

### Topical effects on the level of attention

**Fig 6:**
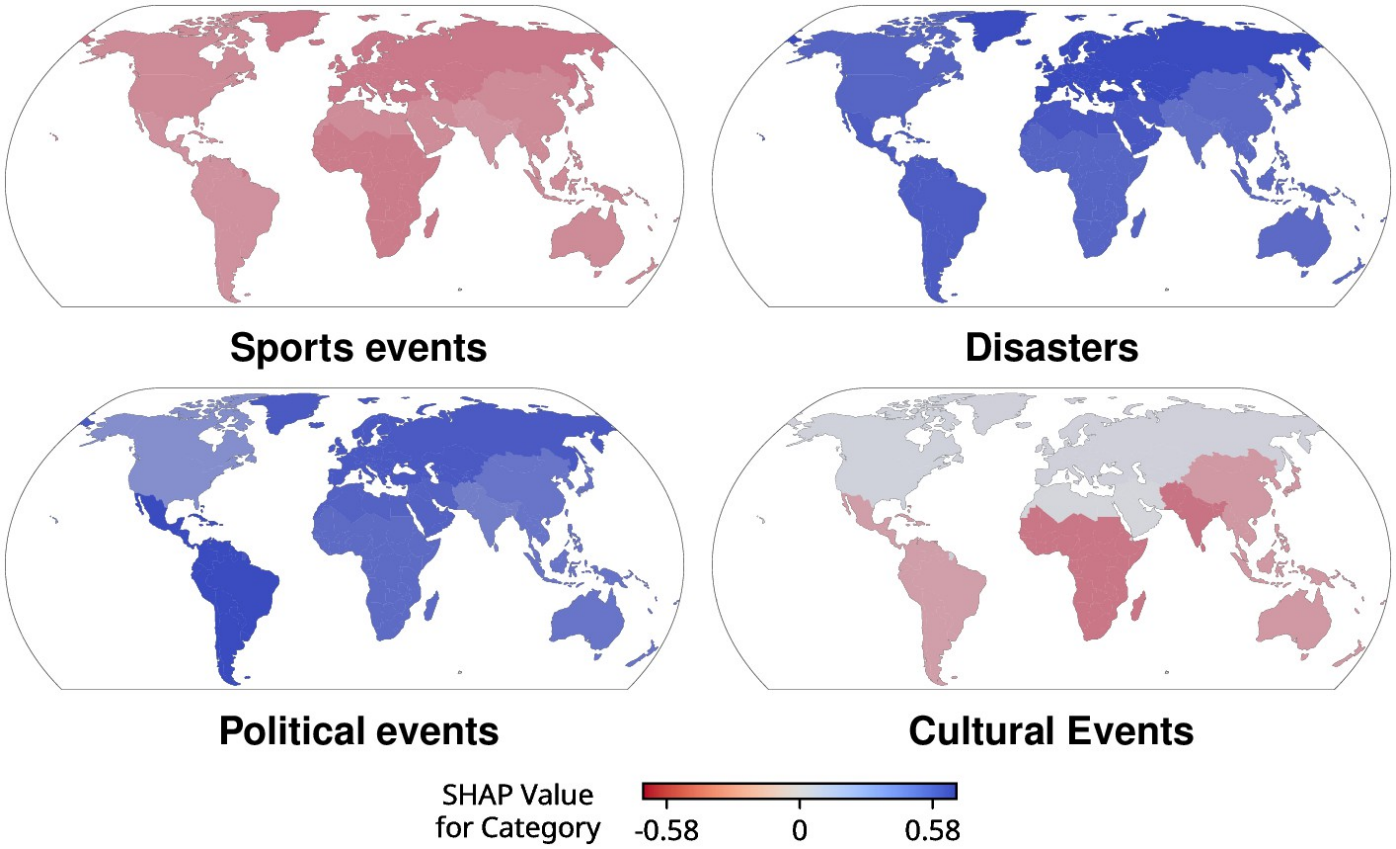
SHAP values for category when predicting continuous attention. We show mean SHAP values for article category by geographic region across all language editions, measuring the influence of category on the predicted views (logarithmic scale). We find that disasters and political events receive more attention than sports and cultural events. Additionally, cultural events in Europe and Central Asia or North America have higher predictions for views than similar events in other regions.

We now closer examine the effect of certain event categories across geographic regions. To this end, we find that article category, according to SHAP values, has a significant effect on the attention articles receive across all language editions (Kruskal-Wallis tests, *p* < 0.0125). When investigating the effect of article category by region for all languages, this significant effect remains across all regions (Kruskal-Wallis tests, *p* < 0.003125), although its magnitude varies by region (Fig 6). For example, political events and disasters generally have a strong positive effect on attention in all language editions (see GitHub for all language-specific figures and S2 Table for pairwise significance tests), and the relative increase for disasters is highest for Europe and Central Asia. Moreover, cultural events located in North America and Europe receive more attention than those in other regions. Finally, we observe lower attention to sports events across all regions, which could be due to the sheer vastness of covered sports leading to an oversaturation of content, which exceeds the capacity of attention that users can devote to Wikipedia.

#### Effects of event-specific features on level of attention

Past research has suggested that certain event-specific factors, e.g., popular teams participating in a sports game [44], can influence attention patterns of an event. We thus now consider a model with features specific to one of our event categories: The effect of death toll on disasters in combination with the wealth of the event country.

**Fig 7:**
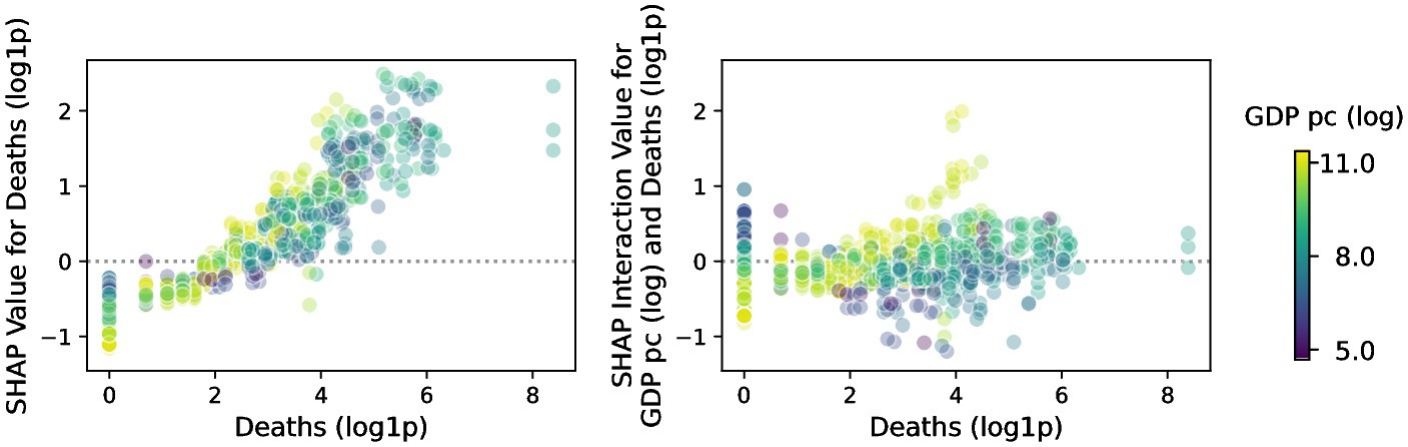
SHAP values and SHAP interaction values for disaster articles. We find that views to disaster articles increase for rising deaths and that this effect is stronger when *GDP*_*pc*_ is high. As we visualize higher *GDP*_*pc*_ with lighter (yellow) and lower values with darker (blue) colors, this finding is observable by the lighter points generally being positioned higher in both plots. Note that *log*1*p*(*x*) = *log*(*x* + 1).

Consequently, we fit an XGBoost regressor predicting views using only deaths and *GDP*_*pc*_ as independent variables (all variables in logarithmic scale). For this regressor, we compute SHAP values and find that attention (i.e., log(views)) increases strongly with higher *GDP*_*pc*_ and deaths (Pearson’s *r* of 0.81 and 0.93, resp.; see Fig 7). Overall, economic power amplifies the effect of event-specific features: the richer the country, the larger the effect of deaths on article attention. For example, even though over 160 people died in Mali during the 2019 Ogossagou massacre (https://w.wiki/Toq), SHAP values for deaths as well as SHAP interaction values for deaths and *GDP*_*pc*_ are lower (1.5 and −0.09, resp.) than for disasters in higher-income countries with fewer deaths. Finally, when considering just disaster articles this simple model using only deaths and *GDP*_*pc*_ performs similarly to the model utilizing the general features described in the previous experiments (mean squared error of 4.85 versus 5.32, no significant difference according to 95% bootstrapped CI). Although we understand that focusing only on a single category might be limiting, for the purpose of this work we use it as a demonstration that more sophisticated, hierarchical models might be necessary to fully explain and determine the exact attention certain events might attract. Given these results, future work should consider both general and event-specific features to predict the load in attention certain events bring to Wikipedia.

## Conclusion

In this work, we set out to investigate how economic and geographic factors skew attention to and coverage of event articles in both larger global and local Wikipedia language editions. For this, we utilize general news values grounded in theory and measure the influence of these factors, primarily *GDP*_*pc*_, on the prediction of machine learning models using SHAP values. We find that the economic power of an event’s country has a linear effect on whether articles receive any coverage or attention at all. In contrast, interpretation of SHAP values for *GDP*_*pc*_ is less straightforward for the regression experiment predicting attention in the form of article views. In this prediction experiment, article category and geographic region emerge as indicators of the attention that particular articles receive across language editions, as for example readers of the Spanish Wikipedia paid higher attention to articles in Latin America as other language editions. Furthermore, we demonstrate the capability of SHAP values to provide local explanations by analyzing outlier events. Overall, we characterize some of the factors that skew attention to Wikipedia, the remediation of which can help address emerging biases and further the Wikimedia Foundation’s quest for knowledge equity and integrity [5]. Hence, by investigating attention and coverage to event articles using news value theory, our findings can serve as groundwork for future research about skews and biases on Wikipedia, which might integrate more sophisticated features and viewpoints according to lessons learned from this analysis. We now discuss limitations of this work and propose avenues for future research.

### Limitations and possibilities for future work

First, although this work considers four Wikipedia editions that cover large parts of the world, our selection excludes regions where other languages are spoken, such as Africa or South-East Asia (e.g., KiSwahili, Arabic, or Hindi). Previous research has shown that behavioral patterns in such smaller or medium-sized language editions can differ considerably from larger editions [38]. In this work, we lacked the language capabilities to analyze these other editions, but in the future we aim to extend our analysis in this direction. Additionally, we make our code available online to enable the application of our methodology to any Wikipedia language edition.

Secondly we use Wikidata to retrieve a candidate list of events. However, Wikidata itself might also be prone to certain biases [58] that could lead to a lack of coverage, which we do not attempt to measure or mitigate. Additionally, we only investigate event items currently linked to a Wikipedia article. Therefore, we could miss effects for events that do not exist on Wikidata or do not have links to valid articles. Thus, although we obtain information from Wikidata as extra-media data [59], we do not consult additional sources to check whether all events are reported. In the future, one could use ground truth databases such as the ACLED database [60] to determine the degree of coverage of events in Wikidata (while acknowledging the biases that may also exist in such databases).

Thirdly, even though we utilize news values and extra-media data for our investigation, we do not examine spillover activity to other articles or language editions, which is an interesting avenue for future work. For example, researchers might also use articles which an event article links to—or vice-versa—as all belonging to a certain event entity [44] or utilize Wikipedia clickstream data [61].

Lastly, attention to Wikipedia can be heavily influenced by media attention. We do however not analyze off-platform news or online search activity for events, as past research suggests that news coverage strongly correlates with online search activity [43, 62], which in turn closely correlates with Wikipedia activity [63].

## Supporting information

S1 Table Significance tests for difference in *GDP*_*pc*_ SHAP values for geographic regions by language and category. We use Mann-Whitney U tests to find differences in *GDP*_*pc*_ SHAP values between articles about events in certain geographic regions within article categories and language editions for the XGBoost Regressor modeling levels of attention. We correct all p-values using the Benjamini-Hochberg procedure and mark significant results as bold.
(PDF)

S2 Table Significance tests for difference in SHAP Values for category across geographic regions by language. We use Mann-Whitney U tests to find differences in Category SHAP values between articles about events in certain geographic regions within article categories and language editions for the XGBoost Regressor modeling levels of attention. We correct all p-values using the Benjamini-Hochberg procedure and mark significant results as bold.
(PDF)
