# Supplementary material for: Poor attention: The wealth and regional gaps in event attention and coverage on Wikipedia
Source: PLoS One. 2023 Nov 8;18(11):e0289325. doi: 10.1371/journal.pone.0289325 (PMC10631632; doi:10.1371/journal.pone.0289325)
Supplement: S1 Table — We use Mann-Whitney U tests to find differences in GDPpc SHAP values between articles about events in certain geographic regions within article categories and language editions for the XGBoost Regressor modeling levels of attention. We correct all p-values using the Benjamini-Hochberg procedure and mark significant results as bold. (PDF) [file pone.0289325.s001.pdf]

Lng	Cat	Region	South Asia	East Asia & Pacific	Latin America & Caribbean	Middle East & North Africa	Europe & Central Asia	Sub-Saharan Africa	North America
German	Culture	**South Asia**	–	–	–	–	–	–	–
		**East Asia & Pacific**		–	–	–	–	–	–
		**Latin America & Caribbean**			–	–	–	–	–
		**Middle East & North Africa**				–	–	–	–
		**Europe & Central Asia**				0.157	–	–	–
		**Sub-Saharan Africa**						–	–
		**North America**				0.156	**0.019**		–
	Disaster	**South Asia**	–	–	–	–	–	–	–
		**East Asia & Pacific**	0.735	–	–	–	–	–	–
		**Latin America & Caribbean**	0.51	0.923	–	–	–	–	–
		**Middle East & North Africa**	0.429	0.243	0.328	–	–	–	–
		**Europe & Central Asia**	0.041	0.17	0.076	**0.001**	–	–	–
		**Sub-Saharan Africa**	0.503	0.823	0.726	0.272	0.449	–	–
		**North America**	**0.01**	0.159	**0.002**	**0.001**	0.892	0.465	–
	Politics	**South Asia**	–	–	–	–	–	–	–
		**East Asia & Pacific**	**0.043**	–	–	–	–	–	–
		**Latin America & Caribbean**	**0.013**	0.387	–	–	–	–	–
		**Middle East & North Africa**	**0.013**	0.778	0.814	–	–	–	–
		**Europe & Central Asia**	**0.001**	**0.034**	**0.005**	0.013	–	–	–
		**Sub-Saharan Africa**	0.821	0.125	0.14	0.069	**<0.001**	–	–
		**North America**	**0.003**	0.864	0.132	0.436	0.104	**0.015**	–
	Sports	**South Asia**	–	–	–	–	–	–	–
		**East Asia & Pacific**	**<0.001**	–	–	–	–	–	–
		**Latin America & Caribbean**	**<0.001**	**<0.001**	–	–	–	–	–
		**Middle East & North Africa**	**<0.001**	0.411	**<0.001**	–	–	–	–
		**Europe & Central Asia**	**<0.001**	**<0.001**	**<0.001**	**<0.001**	–	–	–
		**Sub-Saharan Africa**	0.087	**0.016**	0.331	**0.002**	**<0.001**	–	–
		**North America**	**<0.001**	**0.014**	**<0.001**	0.447	**<0.001**	**<0.001**	–
English	Culture	**South Asia**	–	–	–	–	–	–	–
		**East Asia & Pacific**	**<0.001**	–	–	–	–	–	–
		**Latin America & Caribbean**	**0.001**	**0.001**	–	–	–	–	–
		**Middle East & North Africa**	0.156	0.196	0.156	–	–	–	–
		**Europe & Central Asia**	**<0.001**	0.688	**0.003**	0.296	–	–	–
		**Sub-Saharan Africa**	0.125	**<0.001**	0.065	0.189	**<0.001**	–	–
		**North America**	0.282	**<0.001**	0.01	0.133	**<0.001**	0.197	–
	Disaster	**South Asia**	–	–	–	–	–	–	–
		**East Asia & Pacific**	**<0.001**	–	–	–	–	–	–
		**Latin America & Caribbean**	0.012	**<0.001**	–	–	–	–	–
		**Middle East & North Africa**	0.222	**<0.001**	0.879	–	–	–	–
		**Europe & Central Asia**	**<0.001**	**<0.001**	**<0.001**	**<0.001**	–	–	–
		**Sub-Saharan Africa**	0.013	0.172	0.892	0.219	**<0.001**	–	–
		**North America**	**<0.001**	0.41	**<0.001**	**<0.001**	**<0.001**	0.015	–
	Politics	**South Asia**	–	–	–	–	–	–	–
		**East Asia & Pacific**	**<0.001**	–	–	–	–	–	–
		**Latin America & Caribbean**	**<0.001**	0.27	–	–	–	–	–
		**Middle East & North Africa**	**<0.001**	0.845	0.467	–	–	–	–
		**Europe & Central Asia**	**<0.001**	**<0.001**	**<0.001**	**<0.001**	–	–	–
		**Sub-Saharan Africa**	0.001	**<0.001**	**<0.001**	**<0.001**	**<0.001**	–	–
		**North America**	**<0.001**	**<0.001**	**<0.001**	**0.004**	**<0.001**	**<0.001**	–
	Sports	**South Asia**	–	–	–	–	–	–	–
		**East Asia & Pacific**	**<0.001**	–	–	–	–	–	–
		**Latin America & Caribbean**	**<0.001**	**<0.001**	–	–	–	–	–
		**Middle East & North Africa**	**<0.001**	0.637	**<0.001**	–	–	–	–
		**Europe & Central Asia**	**<0.001**	**<0.001**	**<0.001**	**<0.001**	–	–	–
		**Sub-Saharan Africa**	**<0.001**	0.014	**<0.001**	0.003	**<0.001**	–	–
		**North America**	**<0.001**	**<0.001**	**<0.001**	0.001	**<0.001**	**<0.001**	–
Spanish	Culture	**South Asia**	–	–	–	–	–	–	–
		**East Asia & Pacific**		–	–	–	–	–	–
		**Latin America & Caribbean**		0.609	–	–	–	–	–
		**Middle East & North Africa**				–	–	–	–
		**Europe & Central Asia**		0.036	**<0.001**		–	–	–
		**Sub-Saharan Africa**		0.747	0.777		0.152	–	–
		**North America**		0.522	0.008		**<0.001**	0.84	–
	Disaster	**South Asia**	–	–	–	–	–	–	–
		**East Asia & Pacific**	0.88	–	–	–	–	–	–
		**Latin America & Caribbean**	**<0.001**	**<0.001**	–	–	–	–	–
		**Middle East & North Africa**	0.123	**0.005**	**0.014**	–	–	–	–
		**Europe & Central Asia**	0.241	0.605	**<0.001**	**<0.001**	–	–	–
		**Sub-Saharan Africa**	0.303	0.159	0.189	0.97	**0.009**	–	–
		**North America**	0.324	0.881	**<0.001**	**<0.001**	0.948	**0.022**	–
	Politics	**South Asia**	–	–	–	–	–	–	–
		**East Asia & Pacific**	**0.021**	–	–	–	–	–	–
		**Latin America & Caribbean**	0.21	**<0.001**	–	–	–	–	–
		**Middle East & North Africa**	0.016	0.196	**<0.001**	–	–	–	–
		**Europe & Central Asia**	**<0.001**	**<0.001**	**<0.001**	**<0.001**	–	–	–
		**Sub-Saharan Africa**	0.891	**0.008**	0.053	**0.036**	**<0.001**	–	–
		**North America**	**<0.001**	0.108	**<0.001**	**<0.001**	**<0.001**	**<0.001**	–
	Sports	**South Asia**	–	–	–	–	–	–	–
		**East Asia & Pacific**	**0.001**	–	–	–	–	–	–
		**Latin America & Caribbean**	0.149	**<0.001**	–	–	–	–	–
		**Middle East & North Africa**	**0.014**	**0.075**	**<0.001**	–	–	–	–
		**Europe & Central Asia**	**0.001**	**<0.001**	**<0.001**	**<0.001**	–	–	–
		**Sub-Saharan Africa**	**0.041**	**0.011**	**<0.001**	**0.019**	**<0.001**	–	–
		**North America**	**<0.001**	0.686	**<0.001**	**0.013**	**<0.001**	**<0.001**	–
Italian	Culture	**South Asia**	–	–	–	–	–	–	–
		**East Asia & Pacific**		–	–	–	–	–	–
		**Latin America & Caribbean**			–	–	–	–	–
		**Middle East & North Africa**				–	–	–	–
		**Europe & Central Asia**		0.331			–	–	–
		**Sub-Saharan Africa**						–	–
		**North America**		0.243			**<0.001**		–
	Disaster	**South Asia**	–	–	–	–	–	–	–
		**East Asia & Pacific**	0.129	–	–	–	–	–	–
		**Latin America & Caribbean**	**0.001**	**0.121**	–	–	–	–	–
		**Middle East & North Africa**	0.539	**0.13**	0.686	–	–	–	–
		**Europe & Central Asia**	**<0.001**	0.781	**0.002**	**0.008**	–	–	–
		**Sub-Saharan Africa**	0.32	0.054	**0.002**	0.313	**<0.001**	–	–
		**North America**	**0.001**	0.69	0.053	**0.048**	0.594	**0.002**	–
	Politics	**South Asia**	–	–	–	–	–	–	–
		**East Asia & Pacific**	0.189	–	–	–	–	–	–
		**Latin America & Caribbean**	0.189	**0.008**	–	–	–	–	–
		**Middle East & North Africa**	0.447	0.146	0.547	–	–	–	–
		**Europe & Central Asia**	0.156	0.438	0.006	0.095	–	–	–
		**Sub-Saharan Africa**	0.892	0.082	0.599	0.422	**0.013**	–	–
		**North America**	0.461	0.287	0.828	0.821	**0.022**	0.726	–
	Sports	**South Asia**	–	–	–	–	–	–	–
		**East Asia & Pacific**	0.747	–	–	–	–	–	–
		**Latin America & Caribbean**	0.168	**<0.001**	–	–	–	–	–
		**Middle East & North Africa**	0.449	0.467	**<0.001**	–	–	–	–
		**Europe & Central Asia**	0.214	**<0.001**	**<0.001**	**<0.001**	–	–	–
		**Sub-Saharan Africa**	1	0.93	0.1	1	0.151	–	–
		**North America**	0.97	0.06	0.864	0.892	**<0.001**	0.27	–

Bold = Significant with $p < 0.05$

Empty = No data in either comparison group

Dash = Other half of matrix
