# Supplementary material for: Poor attention: The wealth and regional gaps in event attention and coverage on Wikipedia
Source: PLoS One. 2023 Nov 8;18(11):e0289325. doi: 10.1371/journal.pone.0289325 (PMC10631632; doi:10.1371/journal.pone.0289325)
Supplement: S2 Table — We use Mann-Whitney U tests to find differences in Category SHAP values between articles about events in certain geographic regions within article categories and language editions for the XGBoost Regressor modeling levels of attention. We correct all p-values using the Benjamini-Hochberg procedure and mark significant results as bold. (PDF) [file pone.0289325.s002.pdf]

Language	Region	South Asia	East Asia & Pacific	Latin America & Caribbean	Middle East & North Africa	Europe & Central Asia	Sub-Saharan Africa	North America
German	**South Asia**	–	–	–	–	–	–	–
	**East Asia & Pacific**	**<0.001**	–	–	–	–	–	–
	**Latin America & Caribbean**	**<0.001**	**<0.001**	–	–	–	–	–
	**Middle East & North Africa**	0.403	**<0.001**	**<0.001**	–	–	–	–
	**Europe & Central Asia**	**<0.001**	**0.004**	**<0.001**	**<0.001**	–	–	–
	**Sub-Saharan Africa**	0.974	**<0.001**	**<0.001**	0.223	**<0.001**	–	–
	**North America**	**<0.001**	0.057	0.282	**<0.001**	**<0.001**	**<0.001**	–
English	**South Asia**	–	–	–	–	–	–	–
	**East Asia & Pacific**	**<0.001**	–	–	–	–	–	–
	**Latin America & Caribbean**	**<0.001**	0.555	–	–	–	–	–
	**Middle East & North Africa**	**0.009**	**<0.001**	**<0.001**	–	–	–	–
	**Europe & Central Asia**	**<0.001**	**<0.001**	**<0.001**	**<0.001**	–	–	–
	**Sub-Saharan Africa**	**<0.001**	**<0.001**	**<0.001**	0.449	**<0.001**	–	–
	**North America**	**<0.001**	**0.003**	**<0.001**	**<0.001**	**<0.001**	**<0.001**	–
Spanish	**South Asia**	–	–	–	–	–	–	–
	**East Asia & Pacific**	**<0.001**	–	–	–	–	–	–
	**Latin America & Caribbean**	**0.003**	0.908	–	–	–	–	–
	**Middle East & North Africa**	0.908	**<0.001**	**<0.001**	–	–	–	–
	**Europe & Central Asia**	**<0.001**	**<0.001**	**<0.001**	**<0.001**	–	–	–
	**Sub-Saharan Africa**	0.927	**<0.001**	**<0.001**	0.742	**<0.001**	–	–
	**North America**	**0.001**	**<0.001**	**0.015**	**<0.001**	**<0.001**	**<0.001**	–
Italian	**South Asia**	–	–	–	–	–	–	–
	**East Asia & Pacific**	**0.002**	–	–	–	–	–	–
	**Latin America & Caribbean**	**<0.001**	0.473	–	–	–	–	–
	**Middle East & North Africa**	0.162	**<0.001**	**<0.001**	–	–	–	–
	**Europe & Central Asia**	**0.001**	**<0.001**	**<0.001**	**<0.001**	–	–	–
	**Sub-Saharan Africa**	0.726	**<0.001**	**<0.001**	0.127	**<0.001**	–	–
	**North America**	**0.002**	0.233	**0.002**	**<0.001**	**<0.001**	**<0.001**	–

Bold = Significant with $p < 0.05$
Empty = No data in either comparison group
Dash = Other half of matrix
